# CircRNA hsa_circRNA_0001776 inhibits proliferation and promotes apoptosis in endometrial cancer via downregulating LRIG2 by sponging miR-182

**DOI:** 10.1186/s12935-020-01437-y

**Published:** 2020-08-26

**Authors:** Youjuan Jia, Meijuan Liu, Shuxia Wang

**Affiliations:** 1 Department of Gynecology, Weifang People’s Hospital, 151 Guangwen Street, Kuiwen District, Weifang, 261041 Shandong China; 2 Department of Ultrasound, Yantai Yuhuangding Hospital, Yantai, 264000 Shandong China; 3 Department of Obstetrics, Weifang People’s Hospital, Weifang, 261041 Shandong China

**Keywords:** Endometrial cancer, hsa_circRNA_0001776, miR-182, LRIG2

## Abstract

**Background:**

Endometrial cancer (EC) is a common malignancy of the female reproductive system. Circular RNAs (circRNAs) were demonstrated to exert critical roles in cancers, including EC. This study aimed to investigate the effects of hsa_circRNA_0001776 (circ_0001776) on EC.

**Methods:**

Real-time quantitative PCR (RT-qPCR) was used to measure circ_0001776, microRNA-182 (miR-182) and leucine-rich repeats and immunoglobulin-like domains 2 (LRIG2) expression. The diagnostic and prognostic values of circ_0001776 were identified by receiver operating characteristic (ROC) curve analysis and survival analysis, respectively. RNase R digestion was used to characterize circ_0001776, and the localization of circ_0001776 was evaluated by cell fractionation assay. Then, cell counting kit-8 (CCK-8), colony formation, and flow cytometry analysis were used to detect cell proliferation and apoptosis, respectively. The real-time glycolytic rate (ECAR) and lactate production were measured by extracellular flux analysis and a lactate assay kit, respectively. Bioinformatics analysis and dual-luciferase reporter assay were used to determine the interaction among circ_0001776, miR-182 and LRIG2. The protein expression of LRIG2 was determined by western blot. Moreover, circ_0001776 overexpression vector was used to upregulate circ_0001776 expression in an animal tumor model.

**Results:**

Circ_0001776 and LRIG2 were downregulated, while miR-182 was upregulated in EC tissues and cells. Low expression of circ_0001776 was correlated with the 5-year survival rate of EC patients. Upregulated circ_0001776 markedly attenuated cell proliferation and glycolysis, and enhanced cell apoptosis. Besides, circ_0001776 sponged miR-182 to regulate LRIG2 expression. Circ_0001776 could suppress EC progression by miR-182/LRIG2 axis. Furthermore, we also found that circ_0001776 significantly inhibited tumor growth in vivo.

**Conclusion:**

Our results confirmed that circ_0001776 inhibited EC tumorigenesis and progression via miR-182/LRIG2 axis, providing a potential therapeutic target for EC.

## Highlights

1. Circ_0001776 was lowly expressed in endometrial cancer tissues and cells.
2. Circ_0001776 overexpression inhibited endometrial cancer cell proliferation and glycolysis, and promoted cell apoptosis.
3. Circ_0001776 upregulated LRIG2 expression through targeting miR-182.
4. Circ_0001776 suppressed endometrial cancer progression via miR-182/LRIG2 axis.

## Background

Endometrial cancer (EC), mainly occurred in postmenopausal women, is one of the most common malignancies of the female reproductive system with the incidence of about 1/10,000 in worldwide [1]. EC is conventionally classified into type I (estrogen-dependent) EC and type II (estrogen-nondependent) EC according to molecular genetic features and clinicopathological features, and grades 1 and 2 are regarded as “type I”, while grade 3 is regarded as “type II” [2, 3]. The prognosis of “type I” EC patients was relatively favorable, while “type II” EC was always accompanied by the poor outcomes. However, the early diagnosis of EC was extremely difficult due to the complex uterus endocrine function and the unadvanced technology of anatomy. Thus, it is meaningful to explore the potential targets for EC diagnosis and treatment.

Circular RNAs (circRNAs) are a class of abundant, endogenous, conserved non-coding RNAs, which have a circular structure lacking 3′ poly (A) tails and 5′ caps [4–6]. Altered expression of circRNAs was found in numerous cancers, such as hsa_circ_0001313 in colon cancer [7], circ-BANP in lung cancer [8], hsa_circ_0072995 in breast cancer [8]. Several circRNAs were confirmed to be the ideal biomarkers for the diagnosis, treatment, and prognosis of various human cancers [9, 10]. For example, hsa_circ_0052112 could regulate breast cancer tumorigenesis through facilitating cell metastasis [11]. Additionally, hsa_circRNA_0001776 (circ_0001776) was downregulated in EC tissues [12]. However, the regulatory effects of circ_0001776 in EC remain largely unknown and the underlying mechanisms need further understanding.

MicroRNAs (miRNAs) are a group of small, single-stranded, non-coding RNAs with the length of 18–25 nucleotides, which can be involved in the pathological and physical processes, including cell survival, proliferation and metastasis in various tumors [13–15]. Recent studies showed that circRNAs could sponge miRNAs to exert the regulatory effects [16]. For instance, circ_0044516 could target miR-29a-3p to facilitate cell metastasis in prostate cancer [17]. MicroRNA-182 (MiR-182) was confirmed to aberrantly express in EC [18]. In this research, CircInteractome showed that miR-182 might be a target of circ_0001776, we aimed to explore the functional effects of circ_0001776 and miR-182 on EC tumorigenesis.

Leucine-rich repeats and immunoglobulin-like domains 2 (LRIG2) is a member of LRIG protein family, which harbored a single transmembrane domain [19, 20]. LRIG2 was demonstrated to play a suppressive role in EC [21]. The molecular mechanism of LRIG2 in EC remains unclear. In this study, we predicted that LRIG2 contained the potential binding site of miR-182. Thus, we aimed to explore the functional role of LRIG2 in EC. Mechanically, we investigated the relationship among circ_0001776, miR-182 and LRIG2 and their effects on regulating the tumorigenesis and progression of EC.

## Material and methods

### Tissues samples

Human normal endometrial tissue samples were collected from 30 healthy volunteers, while the tumorous tissues were obtained from 50 endometrial cancer patients who underwent surgery at Weifang People’s Hospital. The clinicopathologic features of these patients were presented in Table 1. All participants signed written informed consent. All experiments in this study were approved by the Human Research Ethics Committee of Weifang People's Hospital.

**Table 1:** Correlation between circ_0001776 expression with clinicopathologic features of EC

Parameters	n	circ_0001776 expression		*P*
		High (n = 25)	Low (n = 25)	
Age (years)				
> 60	31	17	14	0.382
< 60	19	8	11	
Histological stage				
G1 + G2	29	20	9	0.002^**^
G3	21	5	16	
Lymph node metastasis				
Yes	30	11	19	0.021^*^
No	20	14	6	
FIGO stage				
I + II	33	19	14	0.136
III + IV	17	6	11	
ER expression				
Positive	25	10	15	0.116
Negative	25	15	10	
PR expression				
Positive	27	11	16	0.156
Negative	23	14	9	

^*^P < 0.05, ^**^P < 0.01

### Cell lines

RL95-2, Ishikawa, HEC1A, HEC1B and AN3CA cells were human endometrial cancer cell line, while hEEC cells were normal human endometrial epithelial cells. RL95-2, HEC1A and HEC1B cells purchased from American Type Culture Collection (Manassas, VA, USA), and hEEC, Ishikawa and AN3CA cells obtained from Shanghai Zeye Biological Technology Co. Ltd. (Shanghai, China) were used in this study. Dulbecco’s modified Eagle’s medium (DMEM, Gibco, Rockville, MD, USA) containing streptomycin/penicillin (100 U/mL; Invitrogen, Carlsbad, CA, USA) and 10% fetal bovine serum (FBS; Invitrogen) was used to incubate the cells in a humidified chamber, while the cell culture condition was 5% CO_2_ at 37˚C.

### Cell transfection

RL95-2 and Ishikawa cells were selected for the following experiments. Circ_0001776 overexpression vector (circ_0001776) and empty vector (vector), miR-182 mimics (miR-182) and the control (miR-NC), miR-182 inhibitor (anti-miR-182) and the corresponding control (anti-NC), and small interfering RNA (siRNA) targeting LRIG2 (si-LRIG2) and the control group (si-NC) (Genepharma, Shanghai, China) were transfected into RL95-2 and Ishikawa cells by Lipofectamine 3000 Reagent (Invitrogen).

### Real-time quantitative polymerase chain reaction (RT-qPCR)

Total RNA was extracted from non-tumorous tissues and tumor tissues, as well as endometrial cancer cells and hEEC cells using Trizol reagent (Invitrogen), and 2 µg of total RNA was reverse-transcribed using SuperScript III RT (Invitrogen). The specific gene amplified transcript level was measured by a real-time quantitative PCR system. U6 and glyceraldehyde 3-phosphate dehydrogenase (GAPDH) were used to normalize the expression of HK2, miR-182, circ_0001776 and LRIG2, respectively. The specific primer sequences were as follows, circ_0001776: TCAAACCTCGACAAGGTGCT (sense) and CCTTAGAACACCCGGAAGGT (antisense), miR-182: GAGAACAGCAGGTCCAGCAT (sense) and CTTCCTCAGCACAGACCG AG (antisense), LRIG2: CAGTGCATAGCTGGAGGGAGTC (sense) and TACAATGATGAGAAGCTGATTGGCTGCA (antisense), HK2: CAAAGTGACAGTGGGTGTGG (sense) and GCCAGGTCCTTCACTGTCTC(antisense), U6: CTCGCTTCGGCAGCACA (sense) and AACGCTTCACGAATTTGCGT (antisense), GAPDH: AAGGCTGAGAATGGGAAAC (sense) and TTCAGGGACTTGTCATACTTC (antisense). The relative expression of circ_0001776, miR-182 and LRIG2 was assessed by the 2^−∆∆Ct^ method.

### RNase R digestion

2 ug total RNA isolated from RL95-2 and Ishikawa cells were incubated with 6 units of RNase R (Epicenter Biotechnologies, Shanghai, China) for 15 min at 37 °C. After the incubation of total RNA and RNase R, the expression of circ_0001776 was determined through RT-qPCR.

### Subcellular localization

The localization of circ_0001776 was evaluated by cytoplasmic & Nuclear RNA Purification Kit (Norgen Biotek Corp., Belmont, MA, USA). Briefly, cells were lysed by Lysis Buffer J and then centrifuged. Subsequently, the cytoplasmic RNA and nuclear RNA were treated with Buffer SK and anhydrous ethanol, respectively. Then, the nuclear RNA and cytoplasmic RNA were eluted using the spin column. Finally, the expression of circ_0001776 in cytoplasmic and nucleus fractions was detected by RT-qPCR.

### Cell counting kit-8 (CCK-8) assay

The proliferation ability of RL95-2 and Ishikawa cells was assessed by CCK-8 assay. 1 × 10^3^ cells in 200 μL cell suspension were seeded into the 96-well plates and cultured for 24 h. Then, cells were treated with 10 μL CCK-8 solution (Dojindo, Tokyo, Japan) and the absorbance was detected at 24, 48, and 72 h after transfection at 450 nm, respectively.

### Colony formation assay

The selected RL95-2 and Ishikawa cells were plated into the 6-well plates. After incubation for 2 weeks, cells were fixed with 4% paraformaldehyde, stained with 0.4% crystal violet, and counted using a microscope (Bio-Rad Laboratories Inc., Hercules, CA, USA).

### Cell apoptosis assay

The apoptotic cells were detected by flow cytometry analysis using an annexin V-fluorescein isothiocyanate (FITC) apoptosis detection kit (BD Pharmingen, San Diego, CA, USA). In brief, cells were treated with 0.25% trypsin (Gibco), and then centrifuged and removed the supernatant. Subsequently, cells were washed and resuspended with the binding buffer. Then, FITC and propidium iodide (PI) were added in dark for 15 min. Finally, cell apoptosis was assessed by a FACSCalibur flow cytometer (BD Pharmingen).

### Determination of the real-time glycolytic rate (ECAR)

In this study, we used the Seahorse Extracellular Flux Analyzer XF96 (Seahorse Bioscience, North Billerica, MA, USA) to monitor cell metabolic alternation in vitro. After cell transfection, cells with a density of 6 × 10^3^ per well were added into the XF96-well plate and incubated overnight. Then, cells were starved with the non-serum medium for 24 h. For measurement of ECAR, cells were cultured with unbuffered medium, following sequentially injected glucose, oligomycin (OM), and 2-deoxyglucose (2-DG). ECAR detection was noted as mpH/min.

### Determination of lactate

After the transfection of RL95-2 and Ishikawa cells, cells were treated with non-serum medium and starved for 24 h. Then, the cell culture medium was collected to detect the lactate production using a Lactate Assay Kit (KeyGen, Nanjing, China). Lactate production was normalized on the basis of the total protein concentration.

### Dual-luciferase reporter assay

Plasmids carrying the wide-type circ_0001776 (circ_0001776 WT) or mutant-type circ_0001776 (circ_0001776 MUT) were co-transfected with miR-182 mimics or miR-NC in EC cells, while wild-type sequence of LRIG2 3′UTR (LRIG2 WT) or mutant sequence of LRIG2 3′UTR (LRIG2 MUT) plus miR-182 mimics or miR-NC were co-transfected into RL95-2 and Ishikawa cells. After transfection, the relative luciferase activity was detected by the dual-luciferase reporter assay system (Promega, Madison, WI, USA).

### Western blot

Total protein was isolated from the EC tissues and normal tissues, as well as RL95-2 and Ishikawa cells by RIPA lysis buffer (Beyotime, Shanghai, China). Briefly, the total protein was separated using 8–12% odium dodecyl sulfate–polyacrylamide gel electrophoresis, and then the proteins were transferred onto the polyvinylidene difluoride membranes (Sigma, Billerica, MA, USA). Subsequently, the membranes were blocked using 5% non-fat milk and incubated with the primary antibodies against LRIG2 (1:1000, ab157492, Abcam, Cambridge, UK), HK2 (1:1000, ab104836, Abcam) and GAPDH (1:1000, ab8245, Abcam) overnight at 4 °C. Then, the blots were incubated with goat anti-rabbit (1:2000, ab6721, Abcam) or anti-mouse (1:2000, ab205719, Abcam) for 2 h. Finally, the protein signals were calculated by an electrochemiluminescent system (PerkinElmer Life Science, Waltham, MA, USA).

### Mouse xenotransplantation

6 weeks old BALB/c mice were purchased from the Beijing Laboratory Animal Center (Beijing, China). Mice were randomly divided into two groups: vector and circ_0001776. RL95-2 cells stably transfected with circ_0001776 overexpression vector and empty vector were injected into the right flank of the mice. After 7 days, tumor volume was detected every 7 days. After the mice euthanasia on day 28, tumor weight was measured. All the in vivo experiments were approved by Weifang People’s Hospital Experimental Animal Ethics Committee.

### Statistical analysis

All statistical analyses were performed using SPSS 18.0 software. Data were expressed as the mean ± the standard deviations (SD). The interaction between variables was analyzed by Pearson correlation analysis. Student’s *t*-test and one-way ANOVA analysis were performed for comparisons. The diagnosed value was evaluated by the receiver operating characteristic (ROC) curve analysis, and the area under the curve (AUC) < 0.05 meant no diagnostic value. A value of *P* < 0.05 was considered as a statistically significant difference.

## Results

### Circ_0001776 was downregulated in endometrial cancer

**Fig. 1:**
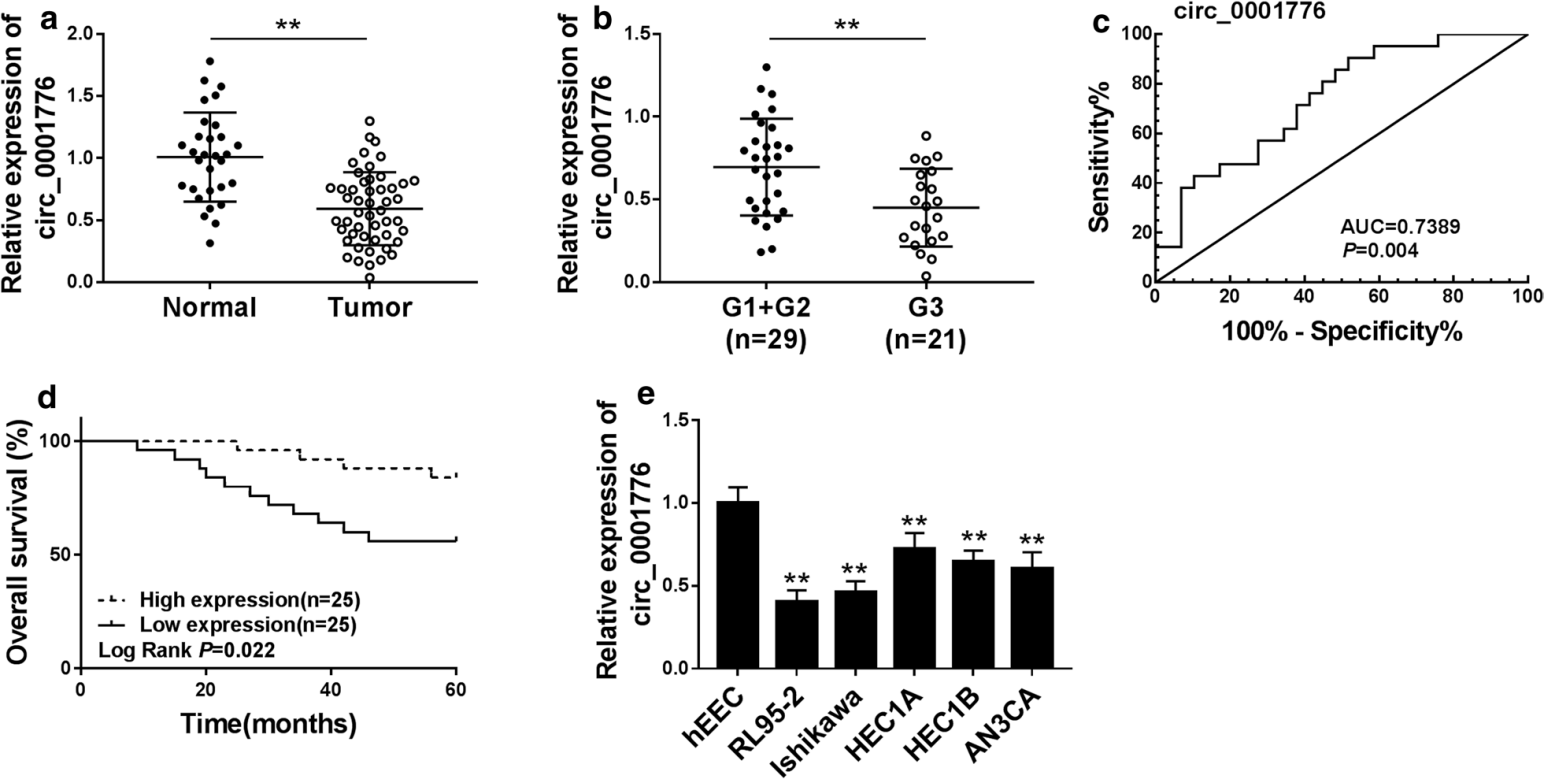
Circ_0001776 was downregulated in endometrial cancer. **a** RT-qPCR was used to detect the expression of circ_0001776 in EC tissues. **b** The expression of circ_0001776 in G1 + G2 and G3 EC tissues was measured by RT-qPCR. **c** The ROC of circ_0001776 as a biomarker was evaluated. **d** Survival was analyzed and compared between patients with high and low levels of circ_0001776 using Kaplan–Meier analysis. **e** The expression of circ_0001776 in EC cells was assessed by RT-qPCR. **P < 0.01

Circ_0001776 expression in EC tissues and cells was measured using RT-qPCR. As shown in Fig. 1a, circ_0001776 was lowly expressed in EC tissues in comparison to that in normal tissues. Compared with the grades in and 2 (G1 + G2) EC tissues, circ_0001776 had a lower expression level in grade 3 (G3) EC tissues (Fig. 1b). Besides, we constructed the ROC curve to explore the potential value of circ_0001776. As described in Fig. 1c, circ_0001776 was confirmed to have the diagnosis value of AUC = 0.7389 (*P* = 0.004). More importantly, we observed that the low expression of circ_0008285 was closely related to the poor survival of EC patients after surgery (Fig. 1d). Furthermore, circ_0001776 expression was decreased in RL95-2, Ishikawa, HEC1A, HEC1B and AN3CA cells relative to that in hEEC cells (Fig. 1e). All these data demonstrated that circ_0001776 was decreased in EC, which might be a biomarker in EC progression.

### Circ_0001776 overexpression suppressed proliferation and facilitated apoptosis of EC cells

**Fig. 2:**
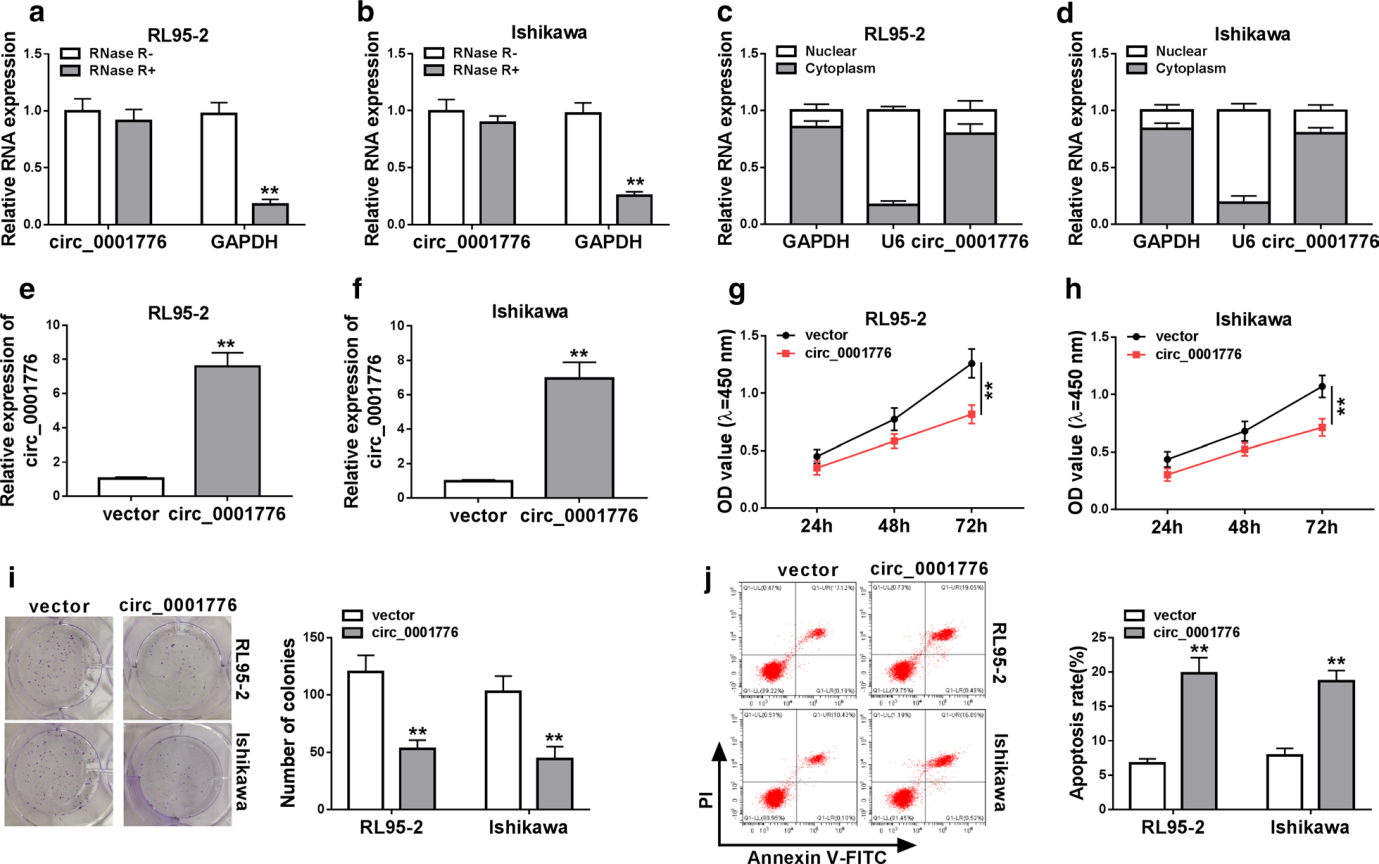
Circ_0001776 overexpression suppressed proliferation and facilitated apoptosis of EC cells. **a**, **b** Circ_0001776 resistance to RNase R was detected by RT-qPCR. **c**, **d** RT-qPCR was used to assess the levels of cytoplasmic control transcript (GAPDH), nuclear control transcript (U6) and circ_0001776 in nuclear and cytoplasmic fractions. **e**, **f** The overexpression efficiency of circ_0001776 was evaluated by RT-qPCR. **g**–**i** Cell proliferation in EC cells transfected with circ_0001776 or vector was detected by CCK-8 assay and colony formation assays. **j** Cell apoptosis in EC cells transfected with circ_0001776 or vector was measured by flow cytometry analysis. **P < 0.01

To elucidate whether circ_0001776 played a key role in EC tumorigenesis, the function assays were performed. Firstly, we confirmed that circ_0001776 was indeed circRNA, which was resistant to RNase R digestion in both RL95-2 and Ishikawa cells (Fig. 2a, b). Then, we detected the subcellular localization of circ_0001776 in EC cells. The results showed that circ_0001776 was mostly located in the cytoplasm of EC cells (Fig. 2c, d). As expected, the expression of circ_0001776 was significantly increased in RL95-2, Ishikawa and hEEC cells transfected with circ_0001776 (Fig. 2e, f and Additional file 1. Fig. S1a). CCK-8 assay indicated that circ_0001776 overexpression markedly inhibited cell proliferation in RL95-2 and Ishikawa cells compared with the control group (Fig. 2g, h). Similarly, we observed the reduction of cell colonies number in RL95-2 and Ishikawa cells transfected with circ_0001776 (Fig. 2i). Additionally, we measured the effects of circ_0001776 overexpression on cell apoptosis. The data suggested that overexpression of circ_0001776 significantly induced cell apoptosis in EC cells (Fig. 2j). Moreover, we detected the protein levels of Cleaved-casp3/total casp3 and Cleaved-casp9/total casp9 in EC cells transfected with vector or circ_0001776. The data showed that circ_0001776 overexpression increased the protein expression of cleaved-casp3 and cleaved-casp9 in EC cells (Additional file 2. Fig. S2a, b). However, circ_0001776 overexpression showed no significant effects on cell proliferation, the number of colonies and apoptosis in HEEC cells (Additional file 1. Fig. S1b–d). These results demonstrated that circ_0001776 was a suppressive circRNA in EC tumorigenesis.

### Circ_0001776 overexpression inhibited the glycolytic metabolism in EC cells

**Fig. 3:**
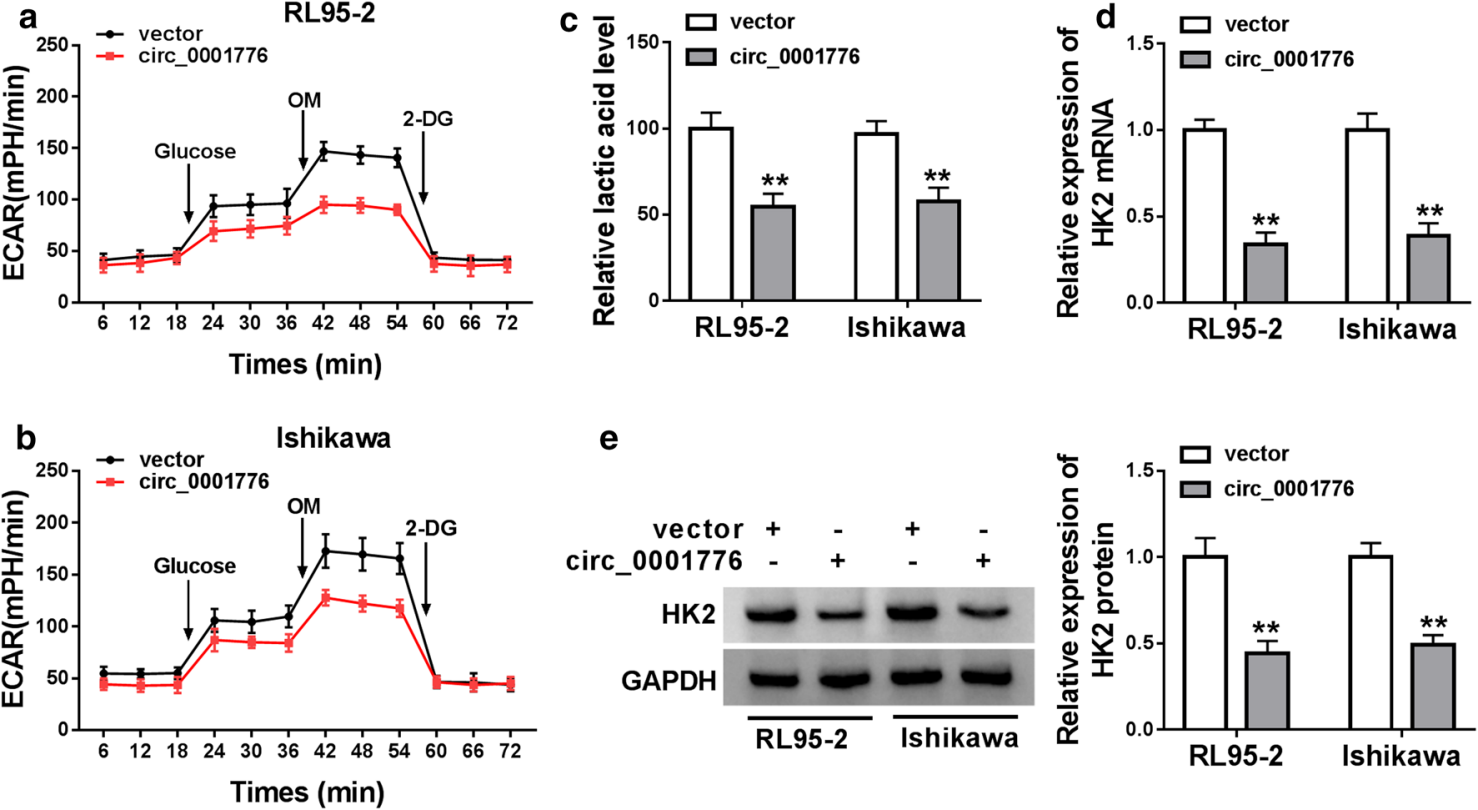
Circ_0001776 overexpression inhibited the glycolytic metabolism in EC cells. **a**, **b** The change of ECAR level with different treatment in RL95-2 and Ishikawa cells was determined after transfecting with control or circ_0001776 overexpression plasmid. **c** The relative lactic acid level in RL95-2 and Ishikawa cells transfected with control or circ_0001776 overexpression plasmid was Examined. **d**, **e** The mRNA and protein expression of HK2 were detected by RT-qPCR and western blot. **P < 0.01

Next, we explored whether overexpression of circ_0001776 could directly affect glycolytic metabolism in EC cells through detecting extracellular acidification rate (ECAR) and lactate2 production. Circ_0001776 upregulation significantly reduced ECAR level in RL95-2 and Ishikawa cells (Fig. 3a, b). Overexpression of circ_0001776 did not change ECAR level in hEEC cells (Additional file 1. Fig. S1E). Moreover, lactic acid production was markedly decreased by circ_0001776 overexpression in RL95-2 and Ishikawa cells (Fig. 3c). We further detected the mRNA and protein expression of HK2 and the data showed that circ_0001776 upregulation significantly suppressed HK expression (Fig. 3d, e). Overall, our data suggested that circ_0001776 could regulate glycolytic metabolism in EC cells.

### Circ_0001776 sponged miR-182

**Fig. 4:**
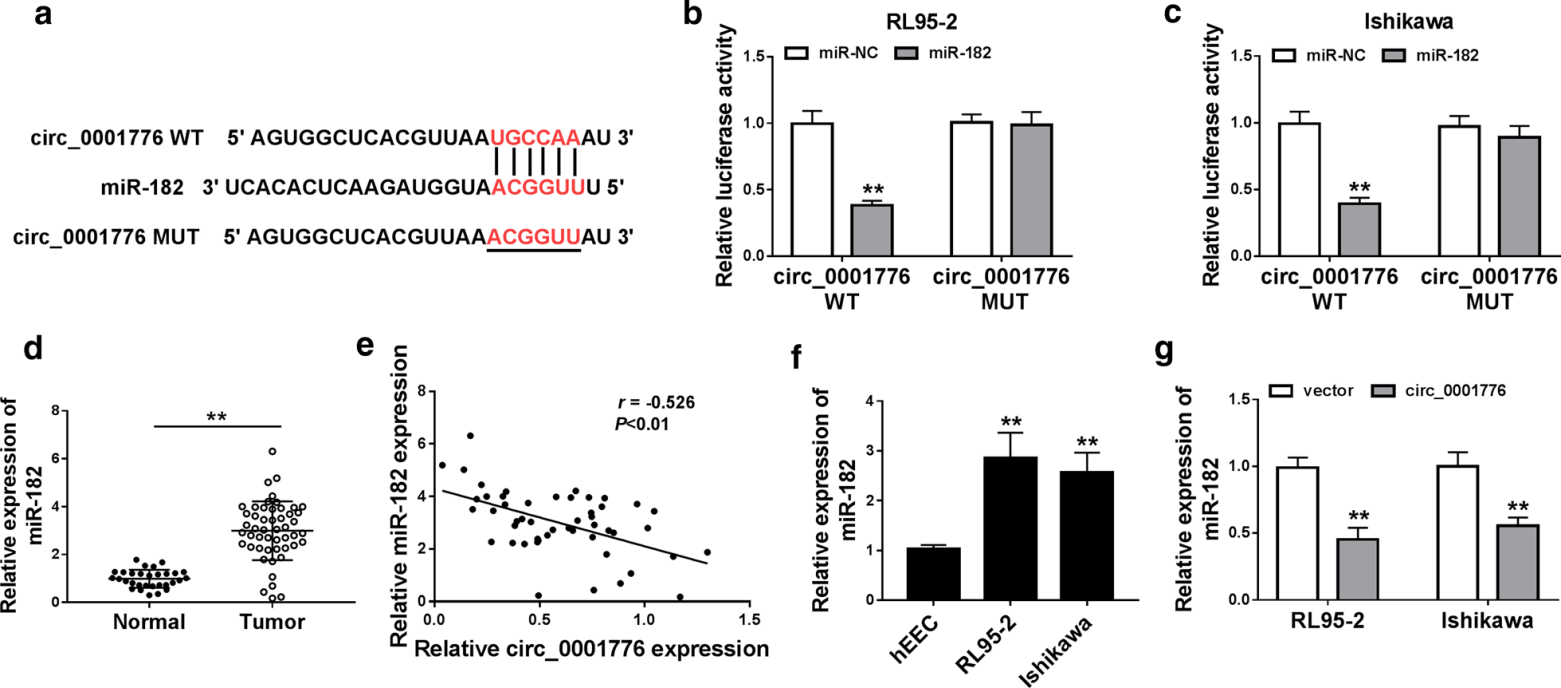
Circ_0001776 sponged miR-182. **a** CircInteractome predicted that miR-182 harbored the binding sites of circ_0001776. **b**, **c** The interaction between circ_0001776 and miR-182 was evaluated by the dual-luciferase reporter assay. **d** The expression of miR-182 in EC tissues was detected by RT-qPCR. **e** The relationship between circ_0001776 and miR-182 was analyzed by Kaplan–Meier analysis (*r* = − 0.526, *P* < 0.01). **f** The expression of miR-182 in EC cells and hEEC cells was examined by RT-qPCR. **g** The expression of miR-182 in EC cells transfected with vector or circ_0001776 was detected by RT-qPCR. **P < 0.01

CircInteractome showed that miR-182 contained the binding sites of circ_0001776 (Fig. 4a). Moreover, the relative luciferase activity was significantly decreased in RL95-2 and Ishikawa cells co-transfected with circ_0001776 WT and miR-182 mimic compared with the control group, while there was no significant change of the luciferase activity in EC cells co-transfected with circ_0001776 MUT and miR-182 mimics (Fig. 4b, c). As shown in Fig. 4d, miR-182 was highly expressed in EC tumor tissues relative to that in normal tissues. Besides, the expression of miR-182 was inversely correlated with circ_0001776 level in EC tissues (Fig. 4e). Consistently, miR-182 level was also facilitated in RL95-2 and Ishikawa cells compared with hEEC cells (Fig. 4f). Moreover, overexpression of circ_0001776 could significantly repress miR-182 expression in EC cells (Fig. 4G). These data revealed that circ_0001776 could serve as a competing endogenous RNA to sponge miR-182.

### Circ_0001776 overexpression inhibited EC progression by sponging miR-182

**Fig. 5:**
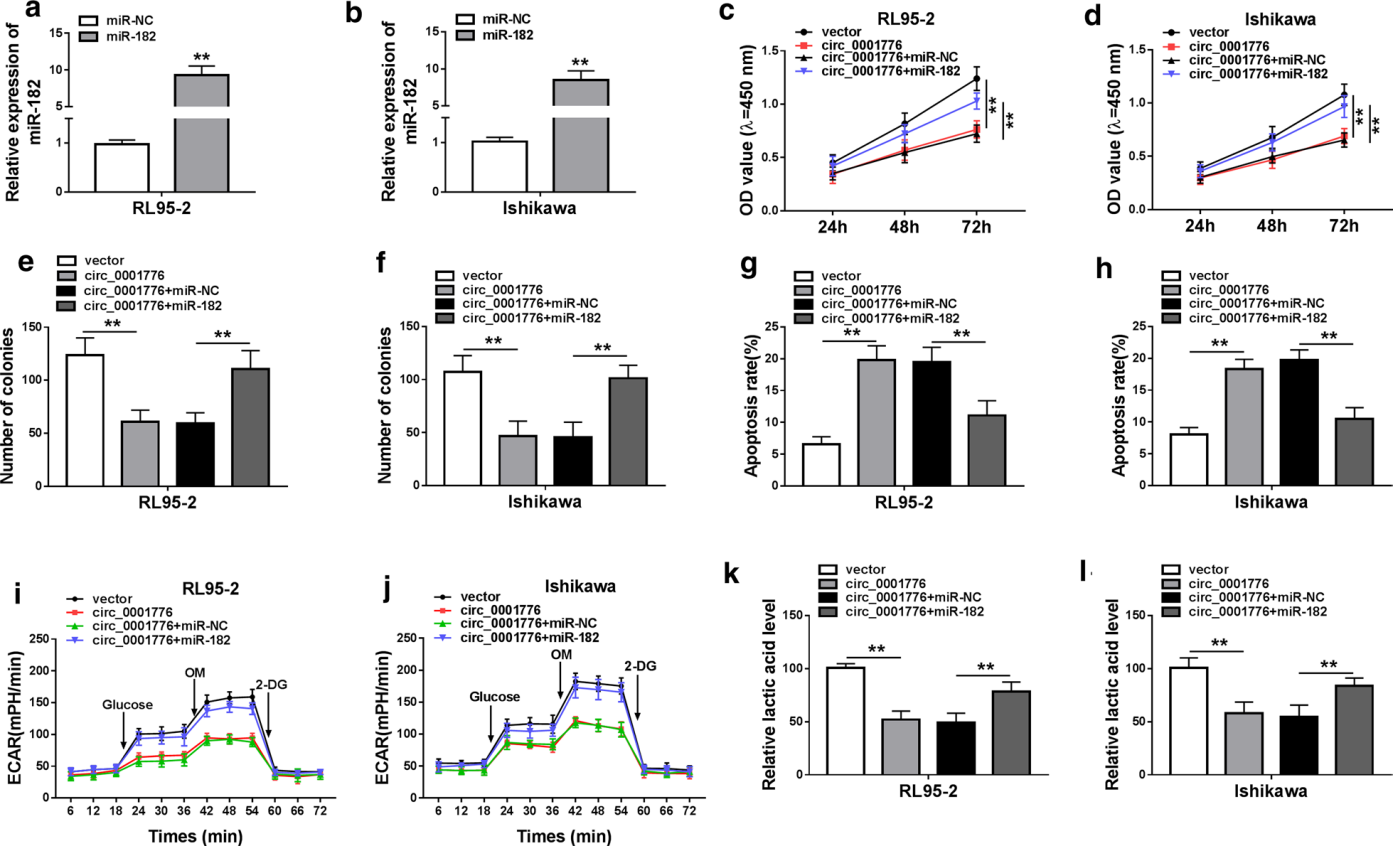
Circ_0001776 overexpression inhibited EC progression by sponging miR-182. **a**, **b** RT-qPCR was used to measure miR-182 expression in RL95-2 and Ishikawa cells transfected with miR-NC or miR-142. **c**, **d** Cell proliferation was measured by CCK-8 assay after transfection. **e**, **f** Cell colony formation assay was used to detect the number of colonies after transfection in EC cells. **g**, **h** Flow cytometry was performed to examine cell apoptosis in RL95-2 and Ishikawa cells after transfection. **i**, **j** ECAR was determined in EC cells after transfection. **k**, **l** The lactic acid level was measured in EC cells after transfection. **P < 0.01

To further investigate whether circ_0001776 exerted its suppressive functions on tumor through miR-182, rescue experiments were performed using miR-182 mimics in EC cells with circ_0001776 overexpression. RT-qPCR demonstrated that miR-182 expression was dramatically increased in EC cells transfected with miR-182 mimics (Fig. 5a, b). Subsequently, we performed the functional experiments in EC cells. CCK-8 assay indicated that the inhibitory effect of circ_0001776 overexpression on cell proliferation was reversed by upregulating miR-182 in both RL95-2 and Ishikawa cells (Fig. 5c, d). We also discovered that miR-182 overexpression significantly rescued the reduction of colonies induced by circ_0001776 overexpression in EC cells (Fig. 5e, f). Flow cytometry analysis indicated that miR-182 upregulation markedly reduced circ_0001776 overexpression-induced cell apoptosis in both RL95-2 and Ishikawa cells (Fig. 5g, h). Furthermore, we evaluated the glycolytic metabolism in EC cells. The data suggested that circ_0001776 upregulation triggered a decrease of glycolytic metabolism, which was reversed by miR-182 overexpression (Fig. 5i, j). Consistent with the results in glycolytic metabolism, the decrease of lactate production in RL95-2 and Ishikawa cells transfected with circ_0001776 was attenuated by upregulating miR-182 (Fig. 5k, l). Taken together, circ_0001776 could inhibit cell proliferation and glycolytic metabolism, and promote cell apoptosis by targeting miR-182 in EC cells.

### LRIG2 was a direct target of miR-182

**Fig. 6:**
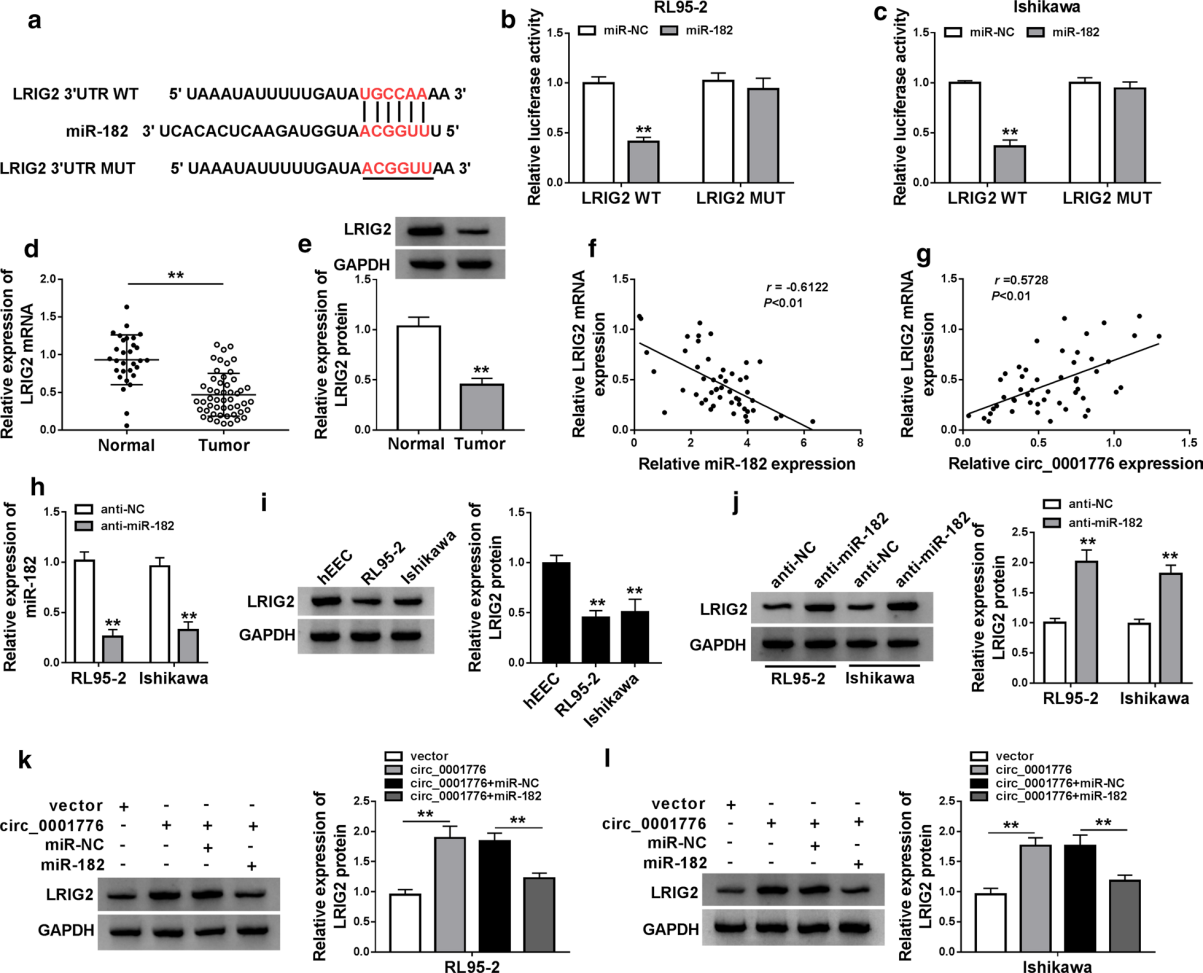
LRIG2 was a direct target of miR-182. **a** The binding site of LRIG2 and miR-182 was predicted by TargetScan. **b**, **c** The relative luciferase activity in EC cells was detected by dual-luciferase reporter assay. **d** The mRNA expression of LRIG2 in EC tissues was examined by RT-qPCR. **e** Western blot was used to detect the protein expression of LRIG2 in EC tissues. **f** The negative relationship between miR-182 and LRIG2 was discovered in EC tissues (*r* = − 0.3122, *P* < 0.01). **g** The positive relationship between circ_0001776 and LRIG2 was observed in EC tissues (*r* = 0.5728, *P* < 0.01). **h** LRIG2 expression was determined after EC cells transfected with anti-NC or anti-miR-182. **i** The protein expression of LRIG2 in EC cells was detected by western blot. **j** LRIG2 expression in EC cells transfected with anti-NC or anti-miR-182 was measured by western blot. **k**, **l** LRIG2 expression in EC cells transfected with vector, circ_0001776, circ_0001776 + miR-NC or circ_0001776 + miR-182 was examined by western blot. **P < 0.01

To investigate the potential molecular mechanism of miR-182 in EC, we screened the potential target gene of miR-182 via TargetScan. As shown in Fig. 6a, LRIG2 harbored the binding sites of miR-182. The luciferase activity was significantly decreased in EC cells co-transfected with LRIG2 WT and miR-182 mimics compared with the control group, while the luciferase activity had no significant change in RL95-2 and Ishikawa cells co-transfected with LRIG2 MUT and miR-182 mimics relative to the corresponding control group (Fig. 6b, c). Moreover, RT-qPCR and western bolt assays showed that the mRNA and protein expression of LRIG2 were dramatically attenuated in tumor tissues relative to that in normal tissues (Fig. 6d, e). Furthermore, LRIG2 expression was negatively related to miR-182 level (Fig. 6f) and positively correlated with circ_0001776 expression (Fig. 6g).

Moreover, the expression of miR-182 was significantly decreased in EC cells transfected with anti-miR-182 (Fig. 6h). We found that the protein expression of LRIG2 was downregulated in EC cells compared with that in hEEC cells (Fig. 6i). As described in Fig. 6j, downregulation of miR-182 could increase LRIG2 protein expression in EC cells. Besides, overexpression of circ_0001776 facilitated the protein expression of LRIG2, while miR-182 overexpression reversed the promotion effect in both RL95-2 and Ishikawa cells (Fig. 6k, l). Overall, miR-182 directly targeted LRIG2, and circ_0001776 could sponge miR-182 to regulate LRIG2 expression in EC cells.

### LRIG2 knockdown reversed the effects of miR-182 downregulation on EC cells

**Fig. 7:**
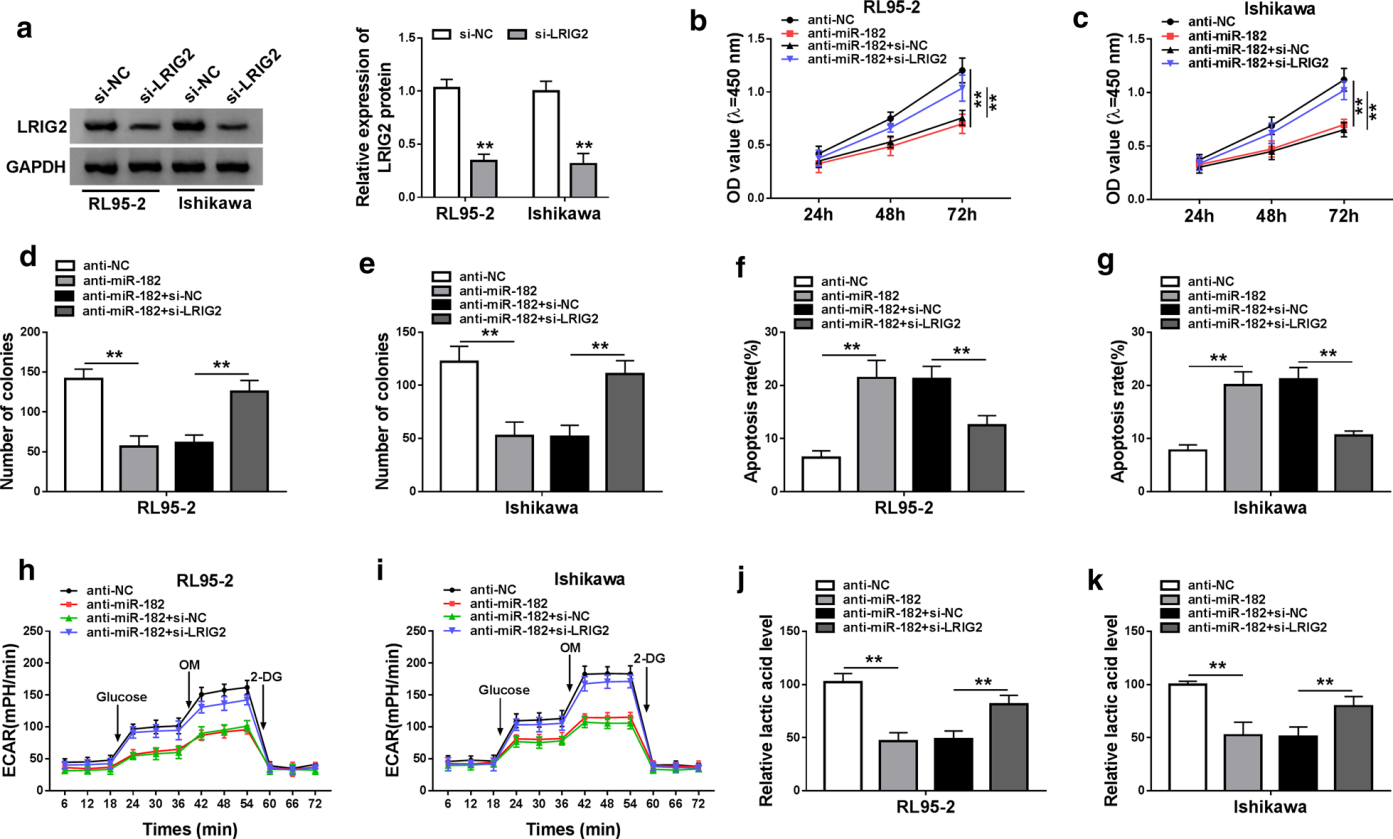
LRIG2 knockdown reversed the effects of miR-182 downregulation on EC progression. **a** The expression of LRIG2 in EC cells transfected with si-NC or si-LRIG2 was determined by western blot. **b**, **c** Cell proliferation was measured in EC cells transfected with anti-NC, anti-miR-182, anti-miR-182 + si-NC and anti-miR-182 + si-LRIG2 by CCK-8 assay. **d**, **e** The number of colonies was assessed in EC cells after transfection. **f**, **g** The apoptotic rate of EC cells after transfection was detected by flow cytometry. **h**, **i** The change of ECRA level in EC cells after transfection was determined. **j**, **k** The lactic acid level in EC cells was examined. **P < 0.01

We detected the knockdown efficiency of LRIG2 in EC cells and observed that transfection of si-LRIG2 could significantly decrease the protein expression of LRIG2 in EC cells (Fig. 7a). As shown in Fig. 7b, c, the suppressive effect of miR-182 downregulation on cell proliferation was blocked by LRIG2 deletion in EC cells. Analogously, the colonies were decreased by downregulating miR-182, while LRIG2 deletion significantly increased the number of colonies in both RL95-2 and Ishikawa cells (Fig. 7d, e). MiR-182 deletion induced cell apoptosis, which was restrained by LRIG2 downregulation in EC cells (Fig. 7f, g). Moreover, the glycolytic metabolism was markedly suppressed in EC cells transfected with anti-miR-182, while LRIG2 deletion could reverse the inhibitory effect of miR-182 deletion on glycolytic metabolism (Fig. 7h, i). Consistently, miR-182 deletion reduced the lactate production in EC cells, while LIRG2 knockdown significantly rescued the reduction of lactate production (Fig. 7j, k). These results indicated that LRIG2 downregulation could block the effects of miR-182 deletion on cell proliferation, apoptosis and glycolytic metabolism in EC cells.

### Circ_0001776 inhibited tumor growth in vivo

**Fig. 8:**
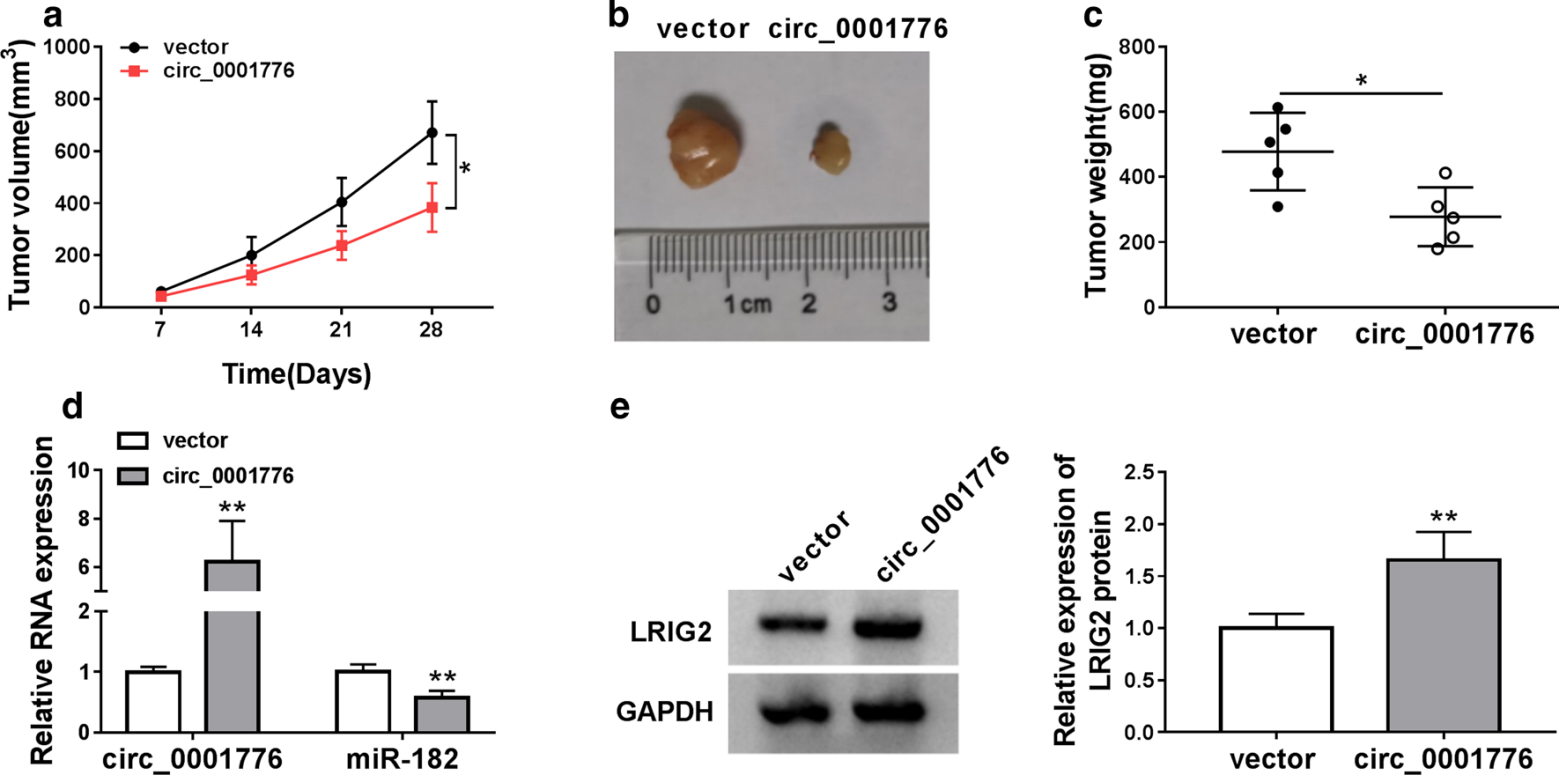
Circ_0001776 inhibited tumor growth in vivo. **a**–**c** The tumor volume and weight were measured in vivo. **d**, **e** The expression of circ_0001776, miR-182 and LRIG2 in the tumor tissues from BALB/c mice was detected using RT-qPCR and western blot, respectively. **P < 0.01

To determine the effects of circ_0001776 on tumor growth in vivo, RL95-2 cells stably transfected with circ_0001776 or vector were injected into the right flank of the mice. The result suggested that circ_0001776 overexpression suppressed tumor growth in vivo (Fig. 8a, b). Furthermore, tumor weight in the circ_0001776 group was significantly lower than that in the control group (Fig. 8c). Besides, we discovered that circ_0001776 expression was upregulated, while miR-182 was downregulated by circ_0001776 overexpression (Fig. 8d). Moreover, the protein expression of LRIG2 was significantly facilitated in the tissues extracted from the mice in circ_0001776 group compared with the control group (Fig. 8e). These data confirmed the suppressive role of circ_0001776 in EC in vivo.

## Discussion

The malignant endometrial tumor is the most common female cancer among all reproductive system diseases, accompanying with increasingly younger women diagnosed with EC [22]. Thus, it is urgent to develop the novel methods for the diagnosis, treatment and prognosis for EC and find out the regulators in the tumorigenesis of EC. Recently, accumulating evidence indicated that circRNAs were closely involved in the regulation of the biological processes in human cancers, including EC [23, 24]. For instance, hsa_circ_0000190 was demonstrated as a novel biomarker for gastric cancer diagnosis, which was significantly associated with lymphatic metastasis, tumor diameter and distal metastasis in gastric cancer [25]. Besides, circ-8073 was confirmed to be a crucial regulator in cell proliferation of endometrial epithelial cells and further targeted miR-449a to regulate the PI3K/AKT/mTOR pathway in endometrial receptivity development [26]. In this research, we found a reduction of circ_0001776 expression in EC tissues and cells. Low expression of circ_0001776 was demonstrated to be related to the grade of EC, and the G3 EC existed lower expression than that in G1 + G2 EC patients. The data in our study were consistent with the results from Ye and his colleagues [12]. Besides, we also observed that EC patients with low circ_0001776 expression had a poor survival rate. Additionally, the functional effects of circ_0001776 were explored by performing functional assays. As expected, overexpression of circ_0001776 significantly inhibited cell growth and glycolytic metabolism, and facilitated cell apoptosis in EC.

Several researches reported that glycolytic metabolism was a key hallmark in human cancers [27]. The activation of glycolysis and the increased lactic acid production were observed in multiple cancer cells, which led to the energy metabolism alteration and were correlated with the prognosis of cancer patients [28]. A recent study confirmed that the glycolytic activity was positively related to EC cell proliferation through activating the MAPK and AMPK/mTOR/S6 pathways [29]. Thus, we detected the effect of circ_0001776 on glycolytic metabolism in EC cells. Our data suggested that circ_0001776 overexpression markedly repressed glycolysis progression in EC cells. All these results pointed out that circ_0001776 expression was a critical factor in the progression of EC.

Previous studies confirmed that circRNAs exerted as the natural sponges of miRNAs and further affected the functions of miRNAs [30]. For example, Chen and his colleagues demonstrated that circ_PTN acted as an oncogenene in the tumorigenesis of glioma through targeting miR-122 to regulate SOX6 expression [31]. A recent research indicated that a circular RNA circ_0025202 sponged miR-182-5p to inhibit cell growth and metastasis in breast cancer [32]. In this study, we demonstrated that miR-182 was a target of circ_0001776 and negatively regulated by circ_0001776 expression. Moreover, the highly expressed miR-182 was observed in EC tissues and cells. Furthermore, our data demonstrated that miR-182 overexpression could reverse the inhibitory effects of circ_0001776 upregulation on cell growth and glycolytic metabolism, and the promotion effect on cell apoptosis in EC cells.

Then, we found that LRIG2 was a target of miR-182. LRIG2 was confirmed to mainly express in the ovaries and uterus among the human organs, which might play an important role in the female reproductive system [33]. Several studies confirmed that LRIG2 exerted the value of prognosis in diverse types of human cancers, including non-small cell lung cancer [34], uterine cervical cancer [35], and hepatocellular carcinoma [36]. Besides, Dae-Shik Suh and his colleagues reported that LRIG2 was a tumor suppressor gene in endometrial adenocarcinoma and inhibited cell growth through regulating PI3K/AKT and EGFR [21]. In our research, we discovered a low expression of LRIG2 in EC tissues and cells. Moreover, LRIG2 expression was negatively regulated by miR-182 level and positively related to the expression of circ_0001776 in EC tissues. Accordingly, miR-182 overexpression could block the promotion effect of circ_0001776 upregulation on LRIG2 expression in EC cells, indicating a circ_0001776-miR-182-LRIG2 regulation network in EC. More function assays suggested that LRIG2 deletion harbored the effects of miR-182 downregulation on cell growth, apoptosis and glycolytic metabolism in EC cells.

## Conclusion

In conclusion, circ_0001776 was identified to be lowly expressed in EC tissues and cells, which might be associated with the grade division and prognosis of EC patients. Functionally, we discovered that circ_0001776 served as a suppressor in the tumorigenesis and progression of EC through enhancing cell apoptosis and inhibiting cell growth and glycolytic metabolism. Collectively, we observed that circ_0001776 was involved in the progression of EC through regulating miR-182/LRIG2 axis and might be a potential biomarker for the prognosis and treatment of EC patients.

## Supplementary information

**Additional file 1: Figure S1** The effect of circ_0001776 overexpression on cell proliferation, apoptosis and ECAR level on hEEC cells.

**Additional file 2: Figure S2** The effect of circ_0001776 overexpression on cell apoptosis.

## Data Availability

The datasets used and/or analyzed during the current study are available from the corresponding author on reasonable request.

## Abbreviations

circRNAs: Endometrial cancer, Circular RNAs
circ_0001776: hsa_circRNA_0001776
miR-182: microRNA-182
RT-qPCR: Real-time quantitative PCR
LRIG2: Immunoglobulin-like domains 2
ROC: Receiver operating characteristic
CCK-8: Cell counting kit-8
miRNAs: microRNAs

## References

[1] Chu D, Wu J, Wang K, Zhao M, Wang C, Li L, Guo R (2018). Effect of metformin use on the risk and prognosis of endometrial cancer: a systematic review and meta-analysis. BMC Cancer.

[2] Bokhman JV (1983). Two pathogenetic types of endometrial carcinoma. Gynecol Oncol.

[3] Di Cristofano A, Ellenson LH (2007). Endometrial carcinoma. Annu Rev Pathol.

[4] Chen LL, Yang L (2015). Regulation of circRNA biogenesis. RNA Biol.

[5] Qu S, Yang X, Li X, Wang J, Gao Y, Shang R, Sun W, Dou K, Li H (2015). Circular RNA: a new star of noncoding RNAs. Cancer Lett.

[6] Kulcheski FR, Christoff AP, Margis R (2016). Circular RNAs are miRNA sponges and can be used as a new class of biomarker. J Biotechnol.

[7] Wang L, Peng X, Lu X, Wei Q, Chen M, Liu L (2019). Inhibition of hsa_circ_0001313 (circCCDC66) induction enhances the radio-sensitivity of colon cancer cells via tumor suppressor miR-338-3p: Effects of cicr_0001313 on colon cancer radio-sensitivity. Pathol Res Pract.

[8] Han J, Zhao G, Ma X, Dong Q, Zhang H, Wang Y, Cui J (2018). CircRNA circ-BANP-mediated miR-503/LARP1 signaling contributes to lung cancer progression. Biochem Biophys Res Commun.

[9] Yu J, Xu QG, Wang ZG, Yang Y, Zhang L, Ma JZ, Sun SH, Yang F, Zhou WP (2018). Circular RNA cSMARCA5 inhibits growth and metastasis in hepatocellular carcinoma. J Hepatol.

[10] Zhang Y, Liu H, Li W, Yu J, Li J, Shen Z, Ye G, Qi X, Li G (2017). CircRNA_100269 is downregulated in gastric cancer and suppresses tumor cell growth by targeting miR-630. Aging.

[11] Zhang HD, Jiang LH, Hou JC, Zhong SL, Zhou SY, Zhu LP, Li J, Wang DD, Sun DW, Ji ZL (2018). Circular RNA hsa_circ_0052112 promotes cell migration and invasion by acting as sponge for miR-125a-5p in breast cancer. Biomed Pharmacother..

[12] Ye F, Tang QL, Ma F, Cai L, Chen M, Ran XX, Wang XY, Jiang XF (2019). Analysis of the circular RNA transcriptome in the grade 3 endometrial cancer. Cancer Manag Res.

[13] Thomson DW, Dinger ME (2016). Endogenous microRNA sponges: evidence and controversy. Nat Rev Genet.

[14] Kawamata T, Tomari Y (2010). Making RISC. Trends Biochem Sci.

[15] Di Leva G, Garofalo M, Croce CM (2014). MicroRNAs in cancer. Annu Rev Pathol.

[16] Han B, Chao J, Yao H (2018). Circular RNA and its mechanisms in disease: From the bench to the clinic. Pharmacol Ther.

[17] Li T, Sun X, Chen L (2019). Exosome circ_0044516 promotes prostate cancer cell proliferation and metastasis as a potential biomarker. J Cell Biochem.

[18] Devor EJ, Schickling BM, Reyes HD, Warrier A, Lindsay B, Goodheart MJ, Santillan DA, Leslie KK (2016). Cullin-5, a ubiquitin ligase scaffold protein, is significantly underexpressed in endometrial adenocarcinomas and is a target of miR-182. Oncol Rep.

[19] Hedman H, Henriksson R (2007). LRIG inhibitors of growth factor signalling - double-edged swords in human cancer?. Eur J Cancer.

[20] Simion C, Cedano-Prieto ME, Sweeney C (2014). The LRIG family: enigmatic regulators of growth factor receptor signaling. Endocr Relat Cancer.

[21] Suh DS, Park SE, Jin H, Lee K, Bae J (2018). LRIG2 is a growth suppressor of Hec-1A and Ishikawa endometrial adenocarcinoma cells by regulating PI3K/AKT- and EGFR-mediated apoptosis and cell-cycle. Oncogenesis.

[22] Zheng R, Zeng H, Zhang S, Chen T, Chen W (2016). National estimates of cancer prevalence in China, 2011. Cancer Lett.

[23] Verduci L, Strano S, Yarden Y, Blandino G (2019). The circRNA-microRNA code: emerging implications for cancer diagnosis and treatment. Mol Oncol.

[24] Xu H, Gong Z, Shen Y, Fang Y, Zhong S (2018). Circular RNA expression in extracellular vesicles isolated from serum of patients with endometrial cancer. Epigenomics.

[25] Chen S, Li T, Zhao Q, Xiao B, Guo J (2017). Using circular RNA hsa_circ_0000190 as a new biomarker in the diagnosis of gastric cancer. Clin Chim Acta..

[26] Liu X, Zhang L, Liu Y, Cui J, Che S, An X, Song Y, Cao B (2018). Circ-8073 regulates CEP55 by sponging miR-449a to promote caprine endometrial epithelial cells proliferation via the PI3K/AKT/mTOR pathway. Biochim Biophys Acta.

[27] Hanahan D, Weinberg RA (2011). Hallmarks of cancer: the next generation. Cell.

[28] Helmlinger G, Sckell A, Dellian M, Forbes NS, Jain RK (2002). Acid production in glycolysis-impaired tumors provides new insights into tumor metabolism. Clin Cancer Res.

[29] Han J, Zhang L, Guo H, Wysham WZ, Roque DR, Willson AK, Sheng X, Zhou C, Bae-Jump VL (2015). Glucose promotes cell proliferation, glucose uptake and invasion in endometrial cancer cells via AMPK/mTOR/S6 and MAPK signaling. Gynecol Oncol.

[30] Panda AC (2018). Circular RNAs Act as miRNA Sponges. Adv Exp Med Biol.

[31] Chen C, Deng L, Nie DK, Jia F, Fu LS, Wan ZQ, Lan Q (2019). Circular RNA Pleiotrophin promotes carcinogenesis in glioma via regulation of microRNA-122/SRY-box transcription factor 6 axis. Eur J Cancer Prev.

[32] Sang Y, Chen B, Song X, Li Y, Liang Y, Han D, Zhang N, Zhang H, Liu Y, Chen T (2019). circRNA_0025202 Regulates Tamoxifen sensitivity and tumor progression via regulating the miR-182-5p/FOXO3a axis in breast cancer. Mol Ther.

[33] Holmlund C, Nilsson J, Guo D, Starefeldt A, Golovleva I, Henriksson R, Hedman H (2004). Characterization and tissue-specific expression of human LRIG2. Gene.

[34] Wang G, Wu J, Song H (2014). LRIG2 expression and prognosis in non-small cell lung cancer. Oncol Lett.

[35] Hedman H, Lindstrom AK, Tot T, Stendahl U, Henriksson R, Hellberg D (2010). LRIG2 in contrast to LRIG1 predicts poor survival in early-stage squamous cell carcinoma of the uterine cervix. Acta Oncol.

[36] Ji D, Wang Y, Li H, Sun B, Luo X (2019). Long non-coding RNA LINC00461/miR-149-5p/LRIG2 axis regulates hepatocellular carcinoma progression. Biochem Biophys Res Commun.

