# Supplementary figures and images for: CircRNA hsa_circRNA_0001776 inhibits proliferation and promotes apoptosis in endometrial cancer via downregulating LRIG2 by sponging miR-182
Source: Cancer Cell Int. 2020 Aug 26;20:412. doi: 10.1186/s12935-020-01437-y (PMC7450557; doi:10.1186/s12935-020-01437-y)

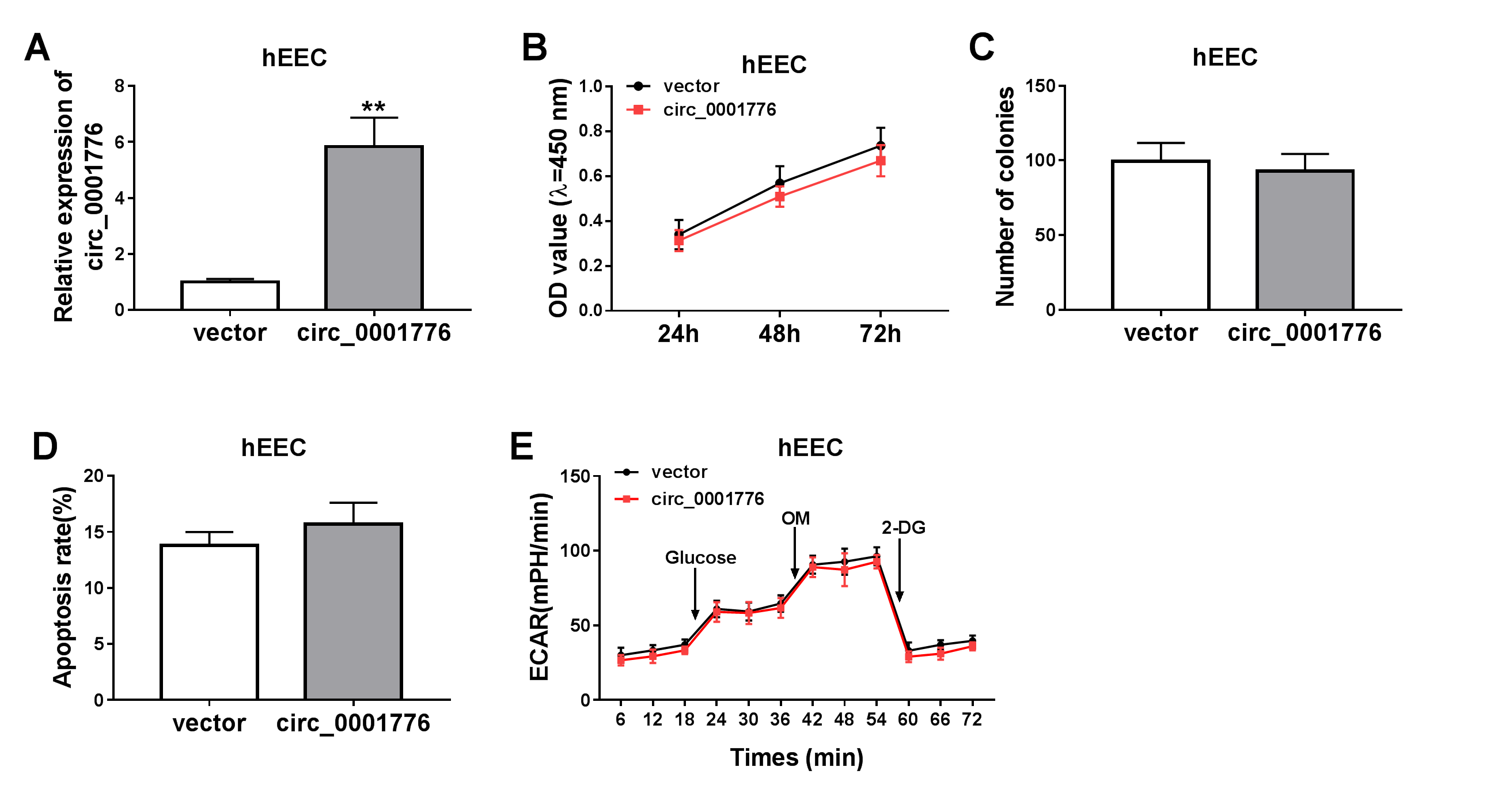

Supplement: Supplementary file 1 — Additional file 1: Figure S1 The effect of circ_0001776 overexpression on cell proliferation, apoptosis and ECAR level on hEEC cells. [file 12935_2020_1437_MOESM1_ESM.tif]

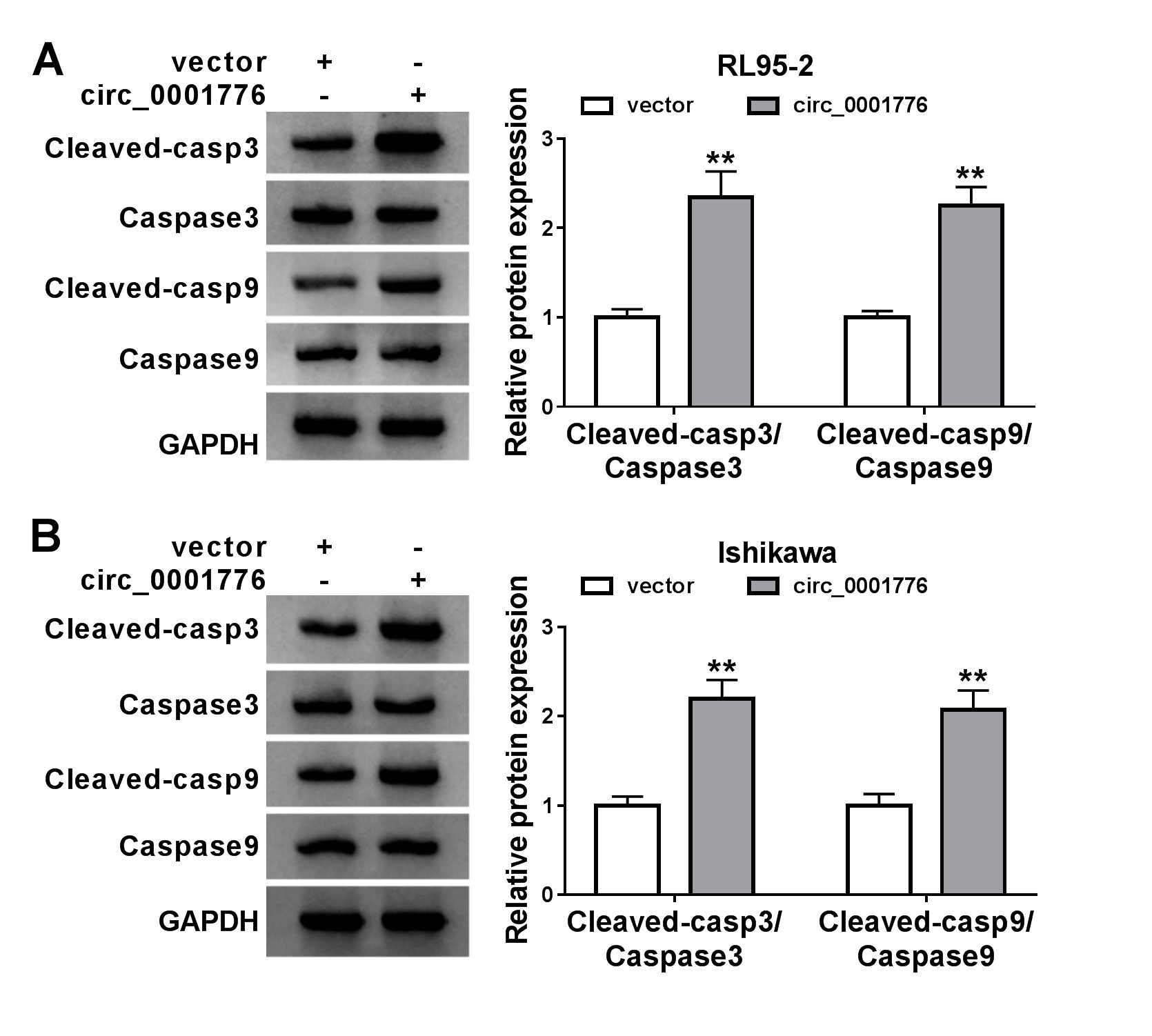

Supplement: Supplementary file 2 — Additional file 2: Figure S2 The effect of circ_0001776 overexpression on cell apoptosis. [file 12935_2020_1437_MOESM2_ESM.tif]
